# On the Educational Treatment of Cretinism

**Published:** 1861-10

**Authors:** J. Mundy

**Affiliations:** of Moravia.


### Art. VII.—ON THE EDUCATIONAL TREATMENT OF CRETINISM.

By J. Mundy, M.D., of Moravia.*

It is well known that the fundamental principle in all attempts to ameliorate the mental condition of cretins is, by judicious efforts, to work on the mind. The question, however, as to whether or not cretinism has ever been influenced by mental impressions, is a subject which has been much neglected in medical discussions. We take it for granted that our readers

---

* In the article on Gheel, from the pen of Dr. Mundy, in the July number of this journal, the author's name, and several other proper names were unfortunately spelled incorrectly. The errors rest, except a misprint or two, with the translator to whom Dr. Mundy had intrusted his manuscript, the author being unavoidably prevented from revising either the translation or the press. The following are the corrections requisite to be made in the paper :—For Sachsenlerge, read *Sachsenberge;* Bull, Orez, *Bull-Ogez;* Pjados, *Pujados ;* Francis, Galt, *Francis Scott;* Veith, *Feith;* Grünty, *Grüntz;* Bulcken, *Bulckens.*

are acquainted with the best historical information on cretinism in all countries; we shall therefore only incidentally touch on the most recent results of the pathological anatomy and physiology of cretinism. It appears to us that the therapeutics of cretinism have as yet obtained but a doubtful support from the statistics of a medically directed education; it is therefore our intention to examine in this article the value of the results so attained. Therapeutics have only a positive value in science when they furnish practical information on progress and cure. Has medical instruction, however, yielded such a result in cretinism? We are inclined absolutely to deny the question of cures, and we ask our readers to examine our views in support of this assertion. Twenty years ago the idea of tending and educating cretins had occurred to but a few individuals in any country. Attempts to cure cretins have only been made in recent times, and these attempts have at last proved *that a cure of this cerebro-spinal disorder is impossible, and that a certain degree of amelioration only is attainable by a system of education under medical supervision.* Have the asserters of the possibility of cure under such circumstances given us sufficient practical proofs of their therapeutics? We find certainly, asserted proofs here and there in books and reviews, but in life and in reality there are none. If men are called upon to cure a disease, they must first understand its etiological diagnosis; but of cretinism and its origin we have not even as yet a correct definition. The etiology of the evil is very vague; the diagnosis is, at the beginning of this disease, very difficult, and in its development without practical value; and medical therapeutics furnish us with nothing to stop its progress, and the system of education pursued presents us with results of but small value. When Dr. Guggenbühl founded his institution, with but scanty means, about fifteen years ago, on the Abendberg, at Interlachen, in Switzerland, for the purpose of nursing and curing cretins, our medical colleagues, as well as the general public of Europe, were charmed with the beautiful but visionary scheme. Many years were requisite before an earnest and temperate examination could prove its futility. What has, we ask, the first founder of this institution done for science and humanity in his especial field? He has done this—First, he has continually kept scientific interest alive to this subject: by which, secondly, several Governments have been incited to establish special asylums for cretins: and thirdly, he has the merit of having been the first who has applied a system of medically directed education to the therapeutics of cretinism. These indisputable merits of Dr. Guggenbühl will, we hope, be always borne in mind by his antagonists in their criticisms on him. The following facts, however, speak against his system:—

1. His asylum* is injudiciously constructed and arranged, especially the new portions of the buildings; appliances for warmth and ventilation, particularly in the dormitories, are almost altogether wanting; and the supply of water is scanty and bad.

2. His general treatment of the patients is arbitrary, and the dietary is unsatisfactory. In the absence of Dr. Guggenbühl, the superintendent and only physician of the asylum (and he is often away an entire month in the winter), no efficient substitute is provided, the patients being left under the charge of a sickly Frenchwoman.

3. The instruction of the children is not worthy of the name of system, and there is a want of those educational appliances without which any good practical result from an institution of this kind cannot be looked for.

During the whole period that Dr. Guggenbühl has directed this asylum, he has never kept even a simple register of cases! The few pamphlets and articles which he has published on his institution have therefore never furnished such positive data and statistical results as science has a right strictly to demand. We must take care not to confound a few *Recitals of Diseases*, which he relates, with the history of cases of cretinism, which he does not furnish. Dr. Guggenbühl has ignored all branches of purely medical detail, and has furnished us with no new statistics of any kind. Dr. Guggenbühl asserts that he has trained many of his patients to become *useful members of society* by his educational system. We have visited two of his patients so designated, but found, unfortunately, that they are still perfect idiots, incapable of making themselves useful, even in the most mechanical occupations of common life. We vainly asked for positive dates respecting other patients reported as progressing towards cure (so weit gebessert). The director of the institution in the Abendberg, has recently applied to the medical faculty and scientific academies of Paris and Petersburg, to compile a statistical report of cretinism of all countries, and to invite discussion on the subject, and these two countries have willingly lent their assistance on this appeal. In Petersburg, as well as in Paris, especial committees have been formed for the purpose of occupying themselves exclusively with this business. Men of the highest renown and talent have become members of this commission in France—such as Brown-Séquard—whilst the Imperial Academy of Medicine at St. Petersburg published, last year, a report of Professor Dr. von Baer on the statistics of cretinism, which we recommend to the especial notice of our readers. From France we shortly expect the report of the commission, which we think will be doubly

* We visited the Abendberg in June, 1861.

interesting, as the revival of the cretin question has been greatly advanced since the annexation of Savoy to France. In addition to this, the Emperor Napoleon III., in strict imitation of his illustrious uncle, had, during his stay at Chambery, in Savoy, last year, a long interview with Parchappe and Niépce, of Grenoble, on cretinism and its cure. In consequence of this imperial conference, a new prize work on this subject has been proposed, and 400,000 francs have been granted to rebuild the lunatic asylum at Bassens, near Chambery, in which there are to be 100 new beds for cretins. The same thing was done by Napoleon I. when, in 1811, he conferred on this subject with Daquin at Chambery, and with Larrey at Paris. A similar prize question was published last year by the German Society for "Psychiatrie und gerichtliche Psychologie;" it was to this effect:—

"Of what good have asylums as yet been to idiotic cretin children? What expectations for the future do they raise? And what arrangements are necessary to increase their utility?"

It is interesting to learn that the only competitor for this prize adopted as his motto the proverb "Nur eitler Hochmuth bricht den Stab über sie;" that he took back his competition work, and that the society has inquired, through the Correspondenzblatt (secretary, Dr. Erlenmayer, at Berndorff, near Coblenz), of its members, whether they desire the original question to stand open for competition for 1862. A great deal has recently been written on the anatomy and physiology of idiotism by Greding, Chiarugi, Romberg, Webster, Engel, Leubuscher, Meier, Ahrens, Kölliker, and finally Virchow, who wrote especially on cretinism. We also possess further valuable information, on this subject, from the pen of Akermann, the brothers Wentzel, Daquin, Ferrûs, Niépce, and Forbes Winslow. We must not omit to mention Rokitansky and Schroeder van der Kolk, those two great men who are not only an honour to Austria and Holland, but also an ornament to the whole world. Valuable contributions to this knowledge have been furnished by Dr. Skae, of Edinburgh, and Dr. Sankey, at Hanwell, who weighed the brains of patients.* And there have been, also, rapid strides in its administrative department. We recall to the memory of our readers the official reports on cretinism issued by the Sardinian and French doctors. In Austria Dr. F. V. Zillner has lately published a very interesting work on idiotic diseases (Jena, 1860). The great experience of this active physician in the Saltzburg district furnishes us with exhaustive proofs of his practical investigations. Dr. Marcher has this year published a handbook of the topography and

* We particularly call the attention of our readers to the distinguished work of Dr. Morel, *Traité des Dégénérescences.*

statistics of the Duchy of Styria, in which he has especially devoted himself to cretinism. It is well known that in Austria, and chiefly in the Salzkammergut and Styria, the so-called endemic cretinism is very prevalent. The experience of these physicians, who are in the daily habit of treating such patients, is of considerable moment. As early as 1855 the meritorious Dr. Köstl, now director of a lunatic asylum at Prague, published a pamphlet on cretinism, as being a proper subject for public care; this was forwarded to the Austrian home minister, Dr. Bach, who placed this, with other memorials, ad acta. The two prizes, which were awarded by the ministry, in 1859, for the best plan for the construction of a cretin asylum, were assigned, the first to the district physician, Dr. Nusser, of Vienna, the second to Dr. Erlenmayer, of Coblenz. In Germany, Dr. Schmidt lately published the report of the cretin institution of Saekingen, in the Grand Duchy of Baden; Dr. Medicus that of the Ecksberg institution, in Bavaria; and lastly, Dr. Zimmer published the eleventh yearly report of the celebrated cretin asylum of Mariaberg, in Wurtemburg. At Hanover a cretin asylum is to be founded by Dr. Davosky; indeed, wherever we look we find such institutions either founded or in a state of construction; thus England, France, Germany, Italy, Switzerland, Austria (Levana), and Russia have all establishments of the kind. In the Social Science Congress at Glasgow, last year, the project of establishing an asylum for idiotic children was discussed, and this year the same congress, at Dublin, renewed it.*

Who does not remember the heartfelt and enlightened letter addressed from Hanwell by Dr. Conolly, on the 17th October, 1860, to Dr. Browne.† If we consider, therefore, all the endeavours which are made for this cause by the most eminent men of Europe, we must come to the inevitable conclusion that they assume it to be a fact proved by experience that cretinism is incurable in the asylums by means of the medical educational system. And at this stage of our case we will again return to the question we raised at the commencement of our article. We have during the last few months visited two of the most distinguished asylums of this kind, namely, the Abendberg, where we had an opportunity of conversing for some time with Dr. Guggenbühl, and the almost princely establishment at Earlswood, near Red Hill, where we had the pleasure of making the acquaintance of Dr. J. Langdon H. Down; besides which we have recently seen the Asylum for

* Even to Gheel, which is not at all a fit place for idiotic children, a Commissioner of the Association of Medical Officers of Asylums for the Insane is to be sent, as decreed at Dublin.

† *Mental Science Journal*, Jan. 1861, page 294.

Idiots at Basle, under the superintendence of Professor Jung; we have also conscientiously examined all the new reports quoted by us on the cretin asylum, and we have not omitted, finally, after a thorough study of the theoretical materials of this question, to obtain practical information by personal examination among the Cretin population as to Saltzburg, Styria, Switzerland, Lower Franconia, and Savoy. We merely quote all this for the single reason that our opinion in regard to the system of medical education and therapeutics of cretinism may not be put down as superficial, or as being obtained by insufficient means; and it must be remembered that an article of this kind written for a journal must be perfectly accurate, and as concise as possible. At the outset we must observe that we are greatly in doubt as to the number of real cretins who are in the asylums especially devoted to them. For instance, we found in the Abendberg among the patients present in June of this year *only one cretin;* the others were merely idiots, two of whom were elderly persons and confirmed lunatics. The number of patients only amounted to twelve, of whom only two paid. Since, the "NaturforscherVersammlung" at Berne, and also several physicians of that city, have given, and that repeatedly, a justly condemnatory verdict against Dr. Guggenbühl's institution, he still continues to uphold this asylum. We do not wish to be misunderstood; we are the first who have described Dr. Guggenbühl's merits in detail, and we consider he has fully earned, and is entitled to, an existence free from pecuniary care, and an honourable acknowledgment of his services. We consider it unjust and unreasonable of his opponents continually to harp on the fact, that he has given no account of the donations which he has received, for we think only the donors themselves have a right to inquire into the subject, and presents given *sine conditione* require no public explanations as to the uses to which they are put. As regards the charges made respecting patients of the higher classes (chiefly English),* who were formerly at the Abendberg, seeking a cure for their malady, they must be considered as a purely private affair, and one into which it is as indiscreet as it is impertinent of his opponents to pry. In science we must divest ourselves of all subjectiveness, but it is a different thing to examine this, or any other institution objectively; it then becomes a sacred duty which we owe not only to truth, but to science and to humanity, to speak fearlessly and openly. We are therefore not satisfied, at the Abendberg, with a few show rooms and the training of the idiots to point out plants, draw a few letters, and stammer some words, nor with the unnecessarily large chapels with stained glass windows,

* We saw two brothers at Earlswood, who were formerly inmates of Abendberg.

which have recently been added, while we found the diet poor, the beds and dormitories bad and dirty, no system of warming and ventilation, a neglect of baths, a want of good drinking-water, and an utter neglect of due care and management in the whole institution. Even its situation is, in our opinion, badly chosen. The reason presumed to justify its selection, namely, that at a certain height above the level of the sea cretinism does not exist among a population, may be termed a reason *ad absurdum*. It must be remembered, too, that the lessened degree pressure of the atmosphere at such a high level, and its dampness, are most injurious, as well as the fogs, the exposure to sun and wind, and the want of good water, added to which is the difficulty of communicating with the asylum in consequence of its position. As we said before, we found at this, the oldest and most celebrated asylum for cretins in the Abendberg, but one cretin, and there is but one to be found at Earlswood, and both these show-specimens of cretinism were pronounced by the directors of the two establishments to be perfectly and hopelessly incurable. Earlswood has a population of idiotic children which varies from 500 to 600, and amongst these there is only *one* cretin. If we promise ourselves a greater number by means of the valueless classification into "semi-cretins" and "cretineux," we can assure our readers that we only found at Earlswood *six* "semi-cretins," whilst at the Abendberg there were none. In the Idiotic Institution at Basle we did not find a single cretin of any one of the three classes among thirty-five idiotic children. It follows, therefore, that the three mentioned institutions can furnish no results of the system of a medically directed education for cretinism, seeing that there are as good as no cretins for cure and education. We have already proved that Dr. Guggenbühl's assertions respecting his former success and his former results are unsatisfactory. Dr. Down, at Earlswood, replied to our inquiry as to the recovery of cretins and idiots, "that there is no probability of being able to educate them so as to fit them for the common purposes of life, and that they can only be made available for the life in the institution." The same opinion has been expressed by other impartial observers in all countries, amongst whom are some of the most distinguished authorities of our science. It is therefore a great error to point out the inadequate management of an institution as the only ground for such insignificant results, and those who imagine that an impartial criticism of this question is actuated by merely subjective objects, and by the desire of opposing and negativing, are still more deceived. It is true that the eleventh Report (1857-8) of the Mariaberg Institution in Wurtemburg maintains that out of fifty-four diseased children, there have been cured in one year three true cretins, three lunatic (irre) cretins, five deaf and dumb idiots, and ten "semi-cretins." But how can we place reliance on such a classification and such results in presence of the Abendberg and Earlswood reports? From 150 patients in different forms of disease which Mariaberg received up to the middle of 1858, fifty-seven are returned as cured! But the few cases which are specially described are treated so vaguely and are sketched so unscientifically, that we cannot divest ourselves of the suspicion that these statements are exaggerated. The yearly report for 1859 of the Cretin Institution of Ecksberg, near Mühldorf in Bavaria, states that from sixty-three patients seven were discharged. It is noticeable, however, that the medical report only mentions two as being cured, and two as convalescent (gebessert), and even these were simple idiots. According to the information imparted by Dr. Macher, of the two Cretin Institutions of Styria—namely, at Admond (with twelve patients) and Gratz (with six), there are no full cretins among them, as none are met there. What does Dr. Zimmer, at Mariaberg, say to this? and also to the assertions of Dr. Zillner, at Salzburg, which are so clear and convincing? and what is the object of these subtle distinctions between idiots and cretins? The opinions which Dr. Kind published* on the occasion of a criticism of Dr. Ziller's important and valuable work are very true; he says that the expressions of sporadic and endemic imbecility (sporadischer und endemischer Blödsinn), classified as idiotism and cretinism, are nothing but tautology. Now we ask, can the question be advanced by discussing imbecility from social causes (idiotism), or from territorial causes (cretinism)? How often can one or other term be properly applied? and what appellation can be given to those who form the largest class, and suffer from both causes combined. The fœtal or early Basiliarsynostose of Virchow has precisely defined cretinism, and he has so considerably modified it that most idiots cannot be termed cretins. Even the division between imbecility from birth (cretinism), or that occurring at a later period (idiotism), cannot be accepted, because, firstly, cretinism would not embrace and exhaust all cases of imbecility from birth; and then, again, there are many cases of acquired imbecility which may be traced back to their fœtal life Generated imbecility is very illusory, as the act of generation is not sufficiently cleared up. Even the division of cretinism, idiotism, and imbecility into three parts, in which the principle of gradation may be observed, rests chiefly on wrong notions of *à priori* origin. This must suffice on the subject of the much-favoured classifications of these diseases. To return, then, to our

* Refer to the *Allgemeine Zeitschrift für Psychiatrie*, vol. xviii., part 1, 1861, p. 89.

original question, we think we have furnished sufficient proofs that the education of cretins, as a therapeutical means, irrespective of its rare application, has never yet produced any cures, nor can ever produce any. How is it possible, we ask, to cure by medical education the following organic changes?

*a* Anomalies of the brain and its membranes and bones of the skull.

*b* Brain atrophy.

*c* Anomalies of the system of bones.

*d* Contractions of muscles, atrophic weakness, fatty degeneration, spasms, and paralysis of muscles.

*e* Goître.

*f* Anomalies of the valves and heart atrophy.

*g* All the anomalies of functions, such as those of the senses, of movement, of feeling, of language and voice, of size, of growth, of carriage of the body, &c., &c.

We will willingly admit that a certain degree of improvement in specified patients is possible, and that it will be practicable in the progress of science and humanity to diminish the *traumatic* congestive, topic, and miasmatic idiotism by progressive culture; but we doubt that science will ever be able successfully to battle against constitutional and incidental idiotism. How little medical pedagogy has till now engaged itself with cretinism in the various institutions, may be proved by the small number of cretins who are their inmates, and the considerable number to be met at large. We are really alarmed by the fact that at a census ordered by Napoleon I., in the year 1811, in the Canton of Valois, in Switzerland, there were found 3000 cretins. (Why does not Dr. Guggenbühl compose any statistical tables of his country?) In the canton of Berne we have the researches of Dr. Schneider; in the Jura formation he counts 1 cretin to every 614 individuals; in the Molass formation, 1 to every 271; and in the Alpine formation, 1 to 361. In the Sardinian States, the census in 1845 proved 7087 cretins and 21,841 goîtres; which gives, in a population of 2,651,106, 1 cretin to 374 individuals, and 1 goître to 121. In some villages the average was 9 cretins in 100. In the total population of Europe, we count, just as in the lunatic population, 1 cretin to every 1000 persons. We think, as we have already maintained of the lunatic population, that this average is below the reality. If, then, the system of medical education has done nothing, and gives no hope for good results in future, the important question arises, "Whether an asylum life for cretins is desirable, and their sequestration justifiable?" As this question forms, at the same time, the great reform debate of the lunatic question, we cannot enter on it in this article. It seems absurd to order a useless sequestration of cretins, as it is unreasonable and unjust. It cannot be denied that, with all the ostentatious display of the cretin schools, they produce no greater results than the outer world. How is it that, with all their means and appliances, they can effect no metamorphosis in the organs of the minds of their patients? The feelings are not capable of exciting any attention in the mind, or of rousing any of its emotions. The mechanical process of making incoherent sounds at which they arrive, the pointing with the hand, and even of writing a well-formed letter, and articulating it aloud, are, after all, nothing but mere tricks, which are far surpassed by apes.

There are some scientific medical men who will not look upon the cretin as a human being, and who maintain that he has no position in the scale of animal creation; they call cretinism not a disease, but an arrest in the formation of an individual, a mere monstrosity of the human race which may be allowed to wither unheeded in the path of life. Far be it from us to share notions so barbarous in their consequences! If we can neither medically nor educationally cure cretinism, let us at least endeavour to minister to these unfortunate beings as much as lies in our power, and ameliorate their condition with all our science and art. We therefore think it a fearful sin if the governments and local authorities of those districts where such unhappy individuals exist neglect drainage and purification in general; such as the regulation of lakes, the position of houses as regards northern aspect, omit to build schools and supply proper medical officers and clergymen, and disregard the improvement of generation by crossing blood in marriage, and do not erect proper nursing establishments for those unfortunates who cannot enjoy their share of earthly good. It may be that there may arise a less prejudiced and more humane time, in which the purely scientific and formal differences of idiotism, cretinism, and imbecility will be altogether ignored in practice. There may be a time when separate asylums will be erected for incurable adult lunatics, and for lunatic children and new cases. These instances ought to be carefully separated from the general lunatic asylums, and not dealt with as is done in these days, where lunatic asylums very much resemble the pattern-card of a commercial traveller, presenting all varieties, from the child in his cradle to the old man on his death-bed. What use this can be to therapeutics we will not here inquire, but only wish this better time may not be too far off to be realized in our own days.

